# *In silico* identification and biochemical characterization of the human dicarboxylate clamp TPR protein interaction network

**DOI:** 10.1002/2211-5463.12521

**Published:** 2018-10-09

**Authors:** Alexandra Bernadotte, Rajnish Kumar, Bengt Winblad, Pavel F. Pavlov

**Affiliations:** ^1^ Department of Molecular Biochemistry and Biophysics Karolinska Institutet Solna Sweden; ^2^ Faculty of Mechanics and Mathematics Lomonosov Moscow State University Russia; ^3^ Division of Neurogeriatrics Department of Neuroscience Care and Society Karolinska Institutet Huddinge Sweden; ^4^ Memory Clinic Theme Aging Karolinska University Hospital Huddinge Sweden

**Keywords:** Alzheimer's disease, dicarboxylate clamp, molecular chaperones, protein interaction network, tetratricopeptide motif, TPR proteins

## Abstract

Dicarboxylate clamp tetratricopeptide repeat (dcTPR) motif‐containing proteins are well‐known partners of the heat shock protein (Hsp) 70 and Hsp90 molecular chaperones. Together, they facilitate a variety of intracellular processes, including protein folding and maturation, protein targeting, and protein degradation. An extreme C‐terminal sequence, the EEVD motif, is identical in Hsp70 and Hsp90, and is indispensable for their interaction with dcTPR proteins. However, almost no information is available on the existence of other potential dcTPR‐interacting proteins. We searched the human protein database for proteins with C‐terminal sequences similar to that of Hsp70/Hsp90 to identify potential partners of dcTPR proteins. The search identified 112 proteins containing a Hsp70/Hsp90‐like signature at their C termini. Gene Ontology enrichment analysis of identified proteins revealed enrichment of distinct protein classes, such as molecular chaperones and proteins of the ubiquitin–proteasome system, highlighting the possibility of functional specialization of proteins containing a Hsp70/Hsp90‐like signature. We confirmed interactions of selected proteins containing Hsp70/Hsp90‐like C termini with dcTPR proteins both *in vitro* and *in situ*. Analysis of interactions of 10‐amino‐acid peptides corresponding to the C termini of identified proteins with dcTPR proteins revealed significant differences in binding strength between various peptides. We propose a hierarchical mode of interaction within the dcTPR protein network. These findings describe a novel dcTPR protein interaction networks and provide a rationale for selective regulation of protein–protein interactions within this network.

**Abbreviations**
AIP: arylhydrocarbon receptor‐interacting protein
Bcl‐G: Bcl‐2‐like protein 14
CHIP: C terminus of HSC70‐interacting protein
dcTPR: dicarboxylate clamp tetratricopeptide repeat
DNAAF2: dynein axonemal assembly factor 2
DNAJC: DnaJ homolog subfamily C
ER: endoplasmic reticulum
FKBP: FK506‐binding protein
GO: Gene Ontology
GST: glutathione‐*S*‐transferase
HRP: horseradish peroxidase
Hsp: heat shock protein
NADSYN1: Nicotinamide adenine dinucleotide synthase 1
PLA: proximity ligation assay
PTGES3: prostaglandin E synthase 3
SGTA: small, glutamine‐rich, tetratricopeptide repeat protein alpha
TPR: tetratricopeptide repeat
Usp19: ubiquitin‐specific peptidase 19

Molecular chaperones are a group of evolutionarily conserved proteins responsible primarily for the maintenance of correct conformation of other protein molecules 1. Furthermore, molecular chaperones are responsible for the intracellular transport and degradation of damaged or misfolded proteins 1. The action of molecular chaperones Hsp70/Hsp90 is mediated by co‐chaperones – proteins interacting with molecular chaperones and providing the specificity of their reactions in the cell 2. Together they form a highly organized network responsible for intracellular protein homeostasis and also enable rapid cell adaptation to various stimuli. The mechanism of interaction of a particular group of co‐chaperones containing the tetratricopeptide repeat (TPR) motif with Hsp70 and Hsp90 is well understood 2. The TPR motif represents a repeat of 34 amino acid residues and consists of seven anti‐parallel α helices 3. Cytosolic Hsp70 and Hsp90 contain the amino acid sequence EEVD at their C termini that mediates their interaction with the TPR domain of co‐chaperones possessing the so called ‘dicarboxylate clamp’ motif – a conserved amino acid sequence enabling efficient binding of two carboxyl groups of C‐terminal aspartic acid and to a lesser extent glutamic acid 3. From 736 TPR motif‐containing proteins annotated in the human UniProt database (http://www.uniprot.org), approximately 25 different proteins interact with Hsp70 and Hsp90 via the dicarboxylate clamp mechanism 2, 4.

To date, very limited information is available on the interactions of dicarboxylate clamp TPR (dcTPR) proteins with proteins other than Hsp70 and Hsp90. The most comprehensive study using unbiased identification of novel interactors focused on the molecular chaperone interactome and included identification of many dcTPR proteins’ partners 5. However, the molecular mechanism of such interactions and involvement of the dcTPR domain were not investigated in this study. Few reports have described details of interactions between proteins containing the acidic C terminus and dcTPR partners via the dicarboxylate clamp mechanism. The mitochondrial protein import receptor Tom20 was shown to interact via its C‐terminal sequence, DDVE, with the dcTPR protein Tom70 6. It was suggested that Tom20 competes with molecular chaperones for binding to Tom70 6. Another dcTPR protein, arylhydrocarbon receptor‐interacting protein (AIP), has also been shown to interact with Tom20 via the dicarboxylate clamp mechanism 7. In yeast, molecular chaperone Hsp104 was shown to interact via its C‐terminal DDLD motif with several dcTPR co‐chaperones under respiratory conditions and this mechanism was suggested to be a part of an adaptation to altered metabolic conditions 8. Recently, the interaction has been described between the dcTPR protein small, glutamine‐rich, tetratricopeptide repeat protein alpha (SGTA) and proteasomal ubiquitin receptor Rpn13 mediated via the C‐terminal sequence DMSLD of Rpn13 9. To understand sequence requirements for acidic peptides to interact with dcTPR proteins, a systematic approach using a phage display has been implemented 10. Biopanning of the T7 random peptide library against the TPR domain of human protein phosphatase 5 revealed a conserved C‐terminal aspartate residue and a less conserved valine/leucine at the −1 position. However, phages that contain MEEVD control sequences corresponding to C termini of Hsp90 were not recovered in this study, indicating a significant limitation of this method for systematic analysis of dcTPR protein–acidic peptide interactions. Molecular determinants for the binding of Hsp70/Hsp90‐like peptides to the TPR1 and TPR2A domains of the dcTPR protein Hop have also been investigated 11. Asp‐0 and Val‐1 of the EEVD motif were identified as general anchor residues, whereas there were individual structural preferences in the C‐terminal peptide sequences for binding to different TPR domains 11.

Two general assumptions can be made on the basis of the data described above. First, molecular chaperones Hsp70 and Hsp90 are not the only proteins able to interact with dcTPR partners. The dicarboxylate clamp mechanism for the protein–protein interactions can be applied to other proteins that contain acidic C termini of similar sequence. Second, due to their rather promiscuous mode of binding, various dcTPR proteins are able to interact with proteins containing C‐terminal acidic peptides structurally related to Hsp70/Hsp90 sequences. To illustrate this, ubiquitin‐specific peptidase 19 (Usp19), in which one of the isoforms contains the MEEVD sequence at the C terminus, was searched for protein interactions using NCBI Gene server (https://www.ncbi.nlm.nih.gov/gene/10869). Many of the listed Usp19 partners identified by various high‐throughput methods, including AIP, DnaJ homolog subfamily C (DNAJC) 7, FK506‐binding protein (FKBP) 52, FKBP36, FKBP38, FKBPL, C terminus of HSC70‐interacting protein (CHIP) and UNC45A, are dcTPR proteins. Yet, none of those interactions nor their biological role has been described in the literature. Many human dcTPR proteins have been implicated in pathogenesis of various disorders ranging from neurodegenerative diseases to cancer, metabolic disorders, asthma, stress‐related disorders, etc. (for selected reviews, see 11, 12, 13, 14, 15, 16). Understanding dcTPR proteins’ function not only as co‐chaperones of Hsp70 and Hsp90 but also as a part of a larger protein–protein interaction network would have important implications in development of new therapies for a wide spectrum of human disorders. In this study, we have explored the network between dcTPR proteins and their partners containing C‐terminal acidic Hsp70/Hsp90‐like sequences.

## Materials and methods

### Sequence alignment and WebLogo presentation

Sequences of 10 C‐terminal amino acids of dcTPR interacting proteins were aligned using the weblogo 3 program version 2.8.2 at http://weblogo.berkeley.edu/.

### Web scrapping and data extraction

A web scrapping processor (written in Python 3, using Requests, urllib2, selenium libraries, Selenium and Firefox browsers) was applied to aggregate data via the BLAST database (https://blast.ncbi.nlm.nih.gov/) and stored in a local database (Postgresql). Data extraction was performed on local servers to filter peptides and proteins with a desired primary structure from the pre‐formed base.

### Gene Ontology enrichment analysis

The identified proteins were subjected to enrichment analysis for GO terms of human proteins using the Gorilla web server [17, 18]. The algorithm identifies the most significant enrichment threshold independently for each GO term in the Human ontology. *P*‐value cutoff was set to < 10 × 10^−4^ and the false discovery rate *q*‐value at 0.01. The significantly enriched GO terms were further summarized using revigo 19 using medium list (0.7) similarity for GO biological process and components.

### Chemicals

All common chemicals were purchased from Sigma‐Aldrich (St Louis, MO, USA) unless stated otherwise.

### Peptides

Peptides > 95% purity were synthesized at Peptide 2.0, Inc. (Chantilly, VA, USA). Peptide sequences were as follows: Hsp90β: NH_2_‐EDASRMEEVD‐COOH; Arg_9_ (R9)–Hsp90β: NH_2_‐RRRRRRRRREDASRMEEVD‐COOH; Hsp70.1: NH_2_‐GSGPTIEEVD‐COOH; Hsp105: NH_2_‐EKNSVNMDLD‐COOH; p23: NH_2_‐SDDEKMPDLE‐COOH; Tom20: NH_2_‐AQSLAEDDVE‐COOH; R9–Tom20: NH_2_‐RRRRRRRRRAQSLAEDDVE‐COOH; DNAAF2: NH_2_‐FQNSLLYDLD‐COOH; Usp19: NH_2_‐PTYSNMEEVD‐COOH; NADSYN1: NH_2_‐AEPQSLDGVD‐COOH; control poly‐D peptide: NH_2_‐DDDDDDDDDD‐COOH. Fluorescein labeling of Hsp90, R9–Hsp90, Tom20 and R9–Tom20 peptides was performed using Lightning‐link^®^ fluorescein labeling kit from Innova Biosciences (Babraham, Cambridge, UK) according to the manufacturer's protocol.

### IMAGE ID for expressed sequence tag clone

The IMAGE ID for expressed sequence tag clones used in this study were as follows: FKBP51: 7969895; AIP: 7480291; Tom70: 3090469; NADSYN1: 5215111; Usp19: 5554022; DNAAF2: 3445311; p23: 7972725; and Bcl‐G: 5215218.

Hsp105 cDNA clone was purchased from RIKEN (Tsukuba, Ibaraki, Japan) (ID: H07D022G08).

### Antibodies

The following antibodies were used: AIP (SAB2105326) and Nicotinamide adenine dinucleotide synthase 1 (NADSYN1) (WH0055191M1) were from Sigma‐Aldrich, FKBP51 (PA1‐020), mouse monoclonal anti‐AIP (MA3‐16515) and p23 (MA3‐414) were from Thermo Fisher Scientific (Uppsala, Sweden), Usp19 (ab68527), dynein axonemal assembly factor 2 (DNAAF2; ab99056) and Hsp105 (ab74174) were from Abcam (Cambridge, MA, USA), Tom 20 (sc‐11415), Tom70 (sc‐390545), Bcl‐2‐like protein 14 (Bcl‐G) (sc‐398223) and mouse monoclonal anti‐FKBP51 (sc‐271547) were obtained from Santa Cruz Biotechnology (Dallas, TX, USA), and Hsp90 (MAB3286) was from R&D Systems (Minneapolis, MN, USA).

### Cloning and purification of human FKBP51, AIP, Usp19, NADSYN1, p23, DNAAF2, Hsp105 and Bcl‐G

Full‐length cDNA clones for respective proteins were obtained from IMAGE consortium (SourceBioScience, Nottingham, UK) and PCR amplified with forward and reverse primers (Table 1) containing overhangs with appropriate restriction sites and cloned into pGEX6 vectors (GE Healthcare, Uppsala, Sweden). Inserts were sequence verified, and the plasmids were transformed into BL21 *Escherichia coli* strain. Glutathione‐*S*‐transferase (GST) fusion proteins were purified from 1 L overnight culture after 2 h of induction with 1 mm IPTG. Cells were pelleted, resuspended in 1× PBS and sonicated 3 × 20 s on ice. EDTA and protease inhibitor cocktail were added to prevent proteolysis. The suspension was centrifuged for 30 min at 50 000 ***g*** to remove cell debris, and supernatant was loaded onto a 1 mL GSTrap™ 4B column (GE Healthcare). After column washing with 30 mL PBS, GST fusion proteins were eluted with 2.5 mL 10 mm glutathione in PBS. Eluate was passed through a PD‐10 column (GE Healthcare) to remove free glutathione. To prepare GST‐tag‐free proteins, we performed overnight cleavage at 4 °C with PreScission protease (GE Healthcare) followed by passage through a GSTrap column to remove free GST protein. Average yield from 1 L starting culture was 1–5 mg of purified proteins. Protein‐containing fractions were collected, checked with SDS/PAGE and kept at −20 °C.

**Table 1:** Primer sequences used in the study

FKBP51 forward	5′‐AAAGGATCCATGACTACTGATGAAGGT‐3′
FKBP51 reverse	5′‐AAACTCGAGTCATACGTGGCCCTCAGG‐3′
AIP forward	5′‐AAAGAATTCATGGCGGATATCATCGCA‐3′
AIP reverse	5′‐AAACTCGAGTCAATGGGAGAAGATCCC‐3′
Tom70_(94–608)_ forward	5′‐AAACCCGGGCAGCGGACACCC‐3′
Tom70_(94–608)_ reverse	5′‐AAAGCGGCCGCTTATAATGTTGGTGGTTT‐3′
Usp19 forward	5′‐AAAGAATTCATGTCTGGCGGGGCCAGT‐3′
Usp19 reverse	5′‐AAACTCGAGCTAATCCACCTCCTCCAT‐3′
Usp19ΔC reverse	5′‐AAACTCGAGTCAGTTGCTGTAGGTGGGGGC‐3′
p23 forward	5′‐AAAGGATCCATGCAGCCTGCTTCTGCA‐3′
p23 reverse	5′‐AAACTCGAGTTACTCCAGATCTGGCAT‐3′
p23ΔC reverse	5′‐AAACTCGAGTCATTTTTCATCATCACTGTC‐3′
DNAAF2 forward	5′‐AAAGTCGACATGGCCAAAGCGGCGGCC‐3′
DNAAF2 reverse	5′‐AAAGCGGCCGCTTAATCCAAATCATATAG‐3′
DNAAF2ΔC reverse	5′‐AAAGCGGCCGCTTACAAAGAATTCTGAAAACT‐3′
NADSYN1 forward	5′‐AAAGTCGACATGGGCCGGAAGGTGACC‐3′
NADSYN1 reverse	5′‐AAAGCGGCCGCTCAGTCCACGCCGTCCAG‐3′
NADSYN1ΔC reverse	5′‐AAAGCGGCCGCTCAGGACTGTGGCTCTGCCCT‐3′

### SDS/PAGE and western blot analysis

Protein (25 μg) was mixed with 2× SDS sample buffer, boiled for 5 min, and loaded onto 4–12% bis‐Tris precast gels (Thermo Scientific, Rockford IL, USA). The samples were electrophoresed and transferred to the nitrocellulose membrane (Whatman, Maidstone, UK), and proteins of interest were detected with specific antibodies using SuperSignal West Pico enhanced chemiluminescence system (Thermo Scientific). Western blot signals were analyzed and quantified using a digital Fujifilm LAS3000 imager (Tokyo, Japan) and las3000 software (Tokyo, Japan).

### Horseradish peroxidase labeling of peptides and dot‐blot experiments

Peptides were dissolved in HEPES‐KOH buffer pH 7.2 at concentration of 1 mg·mL^−1^ and incubated with equimolar amounts of activated horseradish peroxidase (HRP) (Lightning‐link^®^ HRP labeling kit cat. 701‐004, from Innova Biosciences) for 5 min. Reaction was quenched with 0.1 m Tris/HCl, pH 7.5 for 10 min and the products were passed through PD‐10 columns (GE Healthcare). Dot‐blot experiments were performed using GST‐tag‐free proteins applied to the nitrocellulose membrane. After blocking of membrane with 3% BSA in TBS‐T buffer for 1 h and subsequent wash with TBS‐T, HRP‐conjugated peptides (1 × 500 times dilution) were applied and incubated for 1 h. After subsequent washing (3 × 10 min, TBS‐T), membrane was dried and signals were quantified using a digital Fujifilm LAS3000 imager and las3000 software. In competition experiments, increasing concentrations of unlabeled peptides were co‐incubated with peptide–HRP conjugates.

### Cell culture, proximity ligation assay and confocal microscopy

HEK293 cells were cultured in Dulbecco's modified Eagle's medium supplemented with 10% FBS in 5% CO_2_, 95% air at 37 °C. Cells were fixed with 4% paraformaldehyde for 15 min at 25 °C and permeabilized with 0.1% Triton X‐100 for 30 min. A proximity ligation assay (PLA) was conducted essentially according to the manufacturer's instructions (Olink Bioscience). Briefly, HEK293 cells were incubated with the blocking solution Olink Bioscience (Uppsala, Sweden) for 30 min at 37 °C. The cells were incubated overnight at 4 °C with primary rabbit and mouse antibodies forming an assay pair. For negative control experiments, one primary antibody from the pair was omitted. All antibodies were diluted in the antibody diluent provided in the kit. Subsequently, slides were incubated with PLA probes anti‐mouse Minus and anti‐rabbit Plus (Olink Bioscience) for 1 h at 37 °C. Ligation and polymerization steps were performed according to the manufacturer's instructions. After washing twice with PBS, slides were supplied with 4′,6‐diamidino‐2‐phenylindole‐containing mounting medium from Olink Bioscience and samples were visualized using an inverted laser‐scanning confocal microscope (LSM 510 META; Zeiss, Thornwood, NY, USA) equipped with zen 2009 (Carl Zeiss Microimaging GmbH, Jena, Germany) and imagej 1.47 (NIH, Bethesda, MD, USA) software, using a plan‐Neofluar ×40/1.3 oil immersion objective. Quantification of PLA signals was performed using with imagej 1.47 software in five randomly chosen areas (224.8 μm × 224.8 μm) of interest. Student's *t* test, performed with prism software (GraphPad Software Inc., San Diego, CA, USA), was used for statistical analysis of differences in PLA staining upon cells treatment with peptides. *P *< 0.05 was considered to be statistically significant. Data are presented as mean ± SEM.

### Mammalian two‐hybrid system

Tom70 sequence was PCR amplified and subcloned into pACT vector of CheckMate™ mammalian two‐hybrid system (Promega, Madison, WI, USA) using SalI/NotI restriction sites, and Hsp90 and Tom20 DNA sequences were PCR amplified and subcloned into pBIND vector using SalI/NotI restriction sites. Tom70‐pACT/Hsp90‐pBIND and Tom70‐pACT/Tom20‐pBIND were co‐transfected with pLuc plasmid in a 1 : 1 : 1 molar ratio into HEK293 cells using Lipofectamine 3000 transfection reagent (Invitrogen, Carlsbad, CA, USA) according to the manufacturer's protocol. Triplicate samples in 96‐well cell culture plates Corning (Tewskbury, MA, USA) were used in each experiment. Hsp90, R9–Hsp90, Tom20 and R9–Tom20 peptides at concentration 20 μm were added 4 h after transfection. HEK293 cells were cultivated for 48 h, washed twice with PBS and lysed with PBS containing 1% Triton X‐100. Luciferase expression was detected using a luciferase assay kit from BioThema (Huddinge, Sweden) on a 96‐well plate reader from Perkin Elmer (Upplands Väsby, Stockholm, Sweden).

## Results

### Search of human protein database for the proteins containing Hsp70/Hsp90‐like C‐terminal sequences

**Figure 1:**
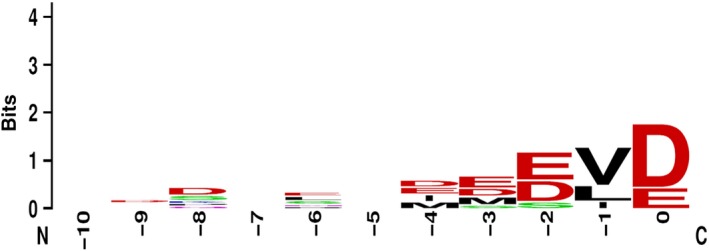
WebLogo presentation of sequence alignment of 10 C‐terminal amino acids from dcTPR‐interacting proteins.

We have previously shown that interactions between the dcTPR protein FKBP51 and peptides resembling C‐terminal sequences of Hsp90 are sequence‐specific and no interaction was observed between FKBP51 and peotide containing a stretch of 10 aspartate residues 20. Here in this study, we systematically searched the human protein database (https://www.ncbi.nlm.nih.gov/protein/) for the protein products containing Hsp70/Hsp90‐like sequence signatures at their C terminus. The alignment sequence logos of 10 utmost C‐terminal amino acids from known dcTPR‐interacting proteins are presented in Fig. 1. Several structural criteria can be extracted to determine the peptide motif for interaction with dcTPR domains. First, the last five amino acids in the sequence are more conserved, a notion supported by the crystallography data where they form most of the contacts within the peptide‐binding groove of the dcTPR domain 2, 20. Second, conserved C‐terminal aspartate or glutamate is required for binding to the dcTPR domain 3. Third, the amino acid adjacent to the last Asp or Glu in the known dcTPR protein partners represents a bulky hydrophobic residue such as Val/Leu/Ile/Phe/Tyr/Trp. Fourth, at least two acidic residues, aspartate or glutamate, need to be present in the analyzed sequence. Finally, we have excluded sequences containing the basic residues lysine and arginine.

**Figure 2:**
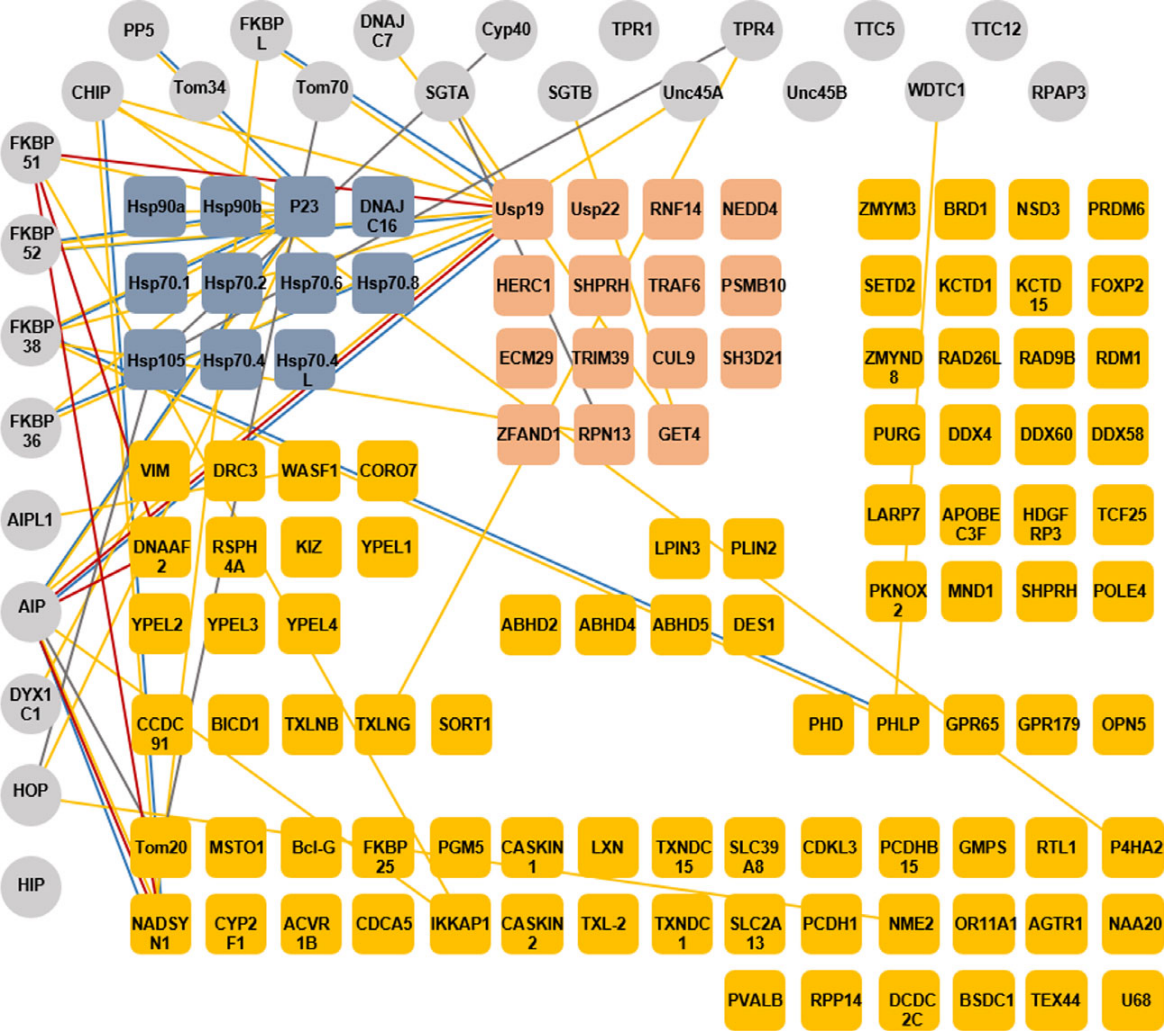
Graphical representation of human proteins with Hsp70/Hsp90‐like C termini identified by global search of the NCBI human protein database. Schematic diagram of interaction network between proteins with Hsp70/Hsp90‐like C termini (in squares) and dcTPR (in circles) proteins. The connections between various proteins are shown by lines. Grey lines show confirmed interactions of proteins other than Hsp70/Hsp90 via dicarboxylate clamp mechanism. Red lines represent connections between proteins via dicarboxylate clamp mechanism established in this study. Blue lines represent protein interactions within network identified by Taipale *et al*. 5. Yellow lines show interactions extracted from protein interaction database (https://www.ncbi.nlm.nih.gov/gene). For simplicity of presentation, we did not show interactions between dcTPR proteins and members of Hsp70 and Hsp90 family.

**Figure 3:**
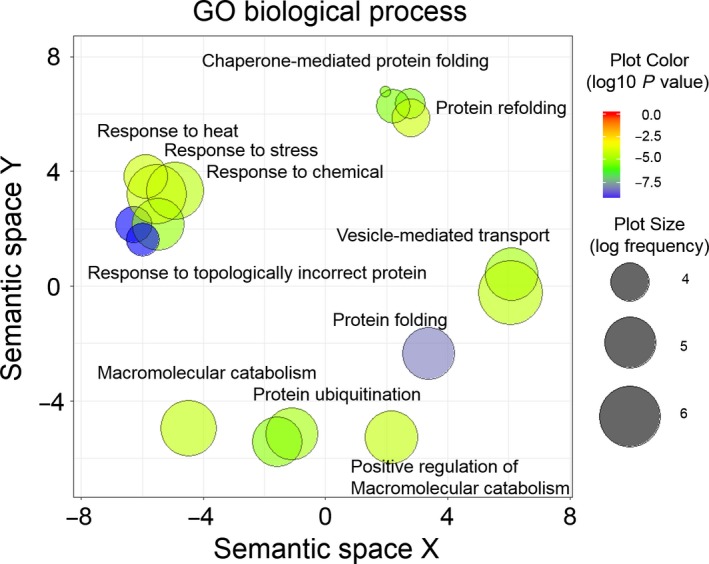
Gene Ontology enrichment analysis. The enrichment analysis for GO terms for human proteins of the identified proteins was performed using Gorilla web server 17, 18 and summarized and visualized by REVIGO 19. Scatter plot generated for ‘GO biological terms’ obtained after redundancy reduction. The plot is colored according to the enrichment log_10_ *P*‐value and the plot size is according to the frequency of the GO term in the database.

The proteins sequences identified in the search were manually inspected with regard to being the full‐length products, presence of potential isoforms and their subcellular localization. Since dcTPR proteins as Hsp70/Hsp90 co‐chaperones are known to reside in the cytosolic compartment and occasionally in the nucleus, we have excluded extracellular, type II membrane proteins or polytopic membrane proteins with their C termini facing the extracellular space or endoplasmic reticulum (ER) lumen, as well as mitochondrial matrix and ER luminal proteins. As a result of the search from the database of > 400K proteins, we have identified 112 human proteins that possess C‐terminal sequences similar to that of Hsp70/Hsp90 molecular chaperones (Table S1). Some of the identified proteins, such as Usp19 or Bcl2‐like protein 14 (BclG), possess the C‐terminal sequence EEVD identical to that of Hsp70/Hsp90, making them highly likely candidates for the interaction with dcTPR proteins. A schematic diagram of the interaction network between the proteins containing Hsp70/Hsp90‐like C termini (shown in squares) and dcTPR (shown in circles) proteins is shown in Fig. 2. Further, we performed enrichment analysis for Gene Ontology (GO) terms of the human proteins using the Gorilla enrichment tool 17, 18. This allowed us to obtain a global overview of the biological processes in which the identified proteins are involved and their cellular location as well. The Gorilla server recognized 104 gene terms out of 112 proteins used as input and identified 100 genes associated with the GO terms. The terms ‘GO biological processes’ showing significant enrichment (*P*‐value < 10^−4^, *q*‐value < 0.01) are listed in Table S2. The significantly enriched terms were clustered and plotted using revigo (Fig. 3). Two distinct functional protein classes were enriched in the general hit population (Fig. 3, Table S2). The first group of proteins represents molecular chaperones and co‐chaperones. They belong to well‐known members of the Hsp70 and Hsp90 family of molecular chaperones as well as to the Hsp105/Hsp110 family of proteins, along with Hsp90 co‐chaperone p23 and Hsp70 co‐chaperone DNAJC16. Biological processes related to protein folding; response to unfolded protein; response to topologically incorrect protein; protein transport; vesicle‐mediated protein transport; response to stress and response to heat encompassing molecular chaperone intracellular functions were significantly enriched with *P*‐values ranging from 4.37 × 10^−10^ to 7.42 × 10^−5^ (Fig. 3, Table S2).

As has been shown in the yeast system, Hsp104 interacts *in vivo* with several dcTPR proteins 8 suggesting that similar type of interactions also exists in human cells. Co‐chaperone p23/prostaglandin E synthase 3 (PTGES3) has been found in complexes with several dcTPR proteins including AIP, CHIP, FKBP36, FKBP38, FKBP51, FKBP52 and PP5 5; however, it is not clear whether these interactions are mediated by the dicarboxylate clamp mechanism or not. No information is available on the interaction of DNAJC16 with dcTPR proteins. The second functional group (~ 13%) represents proteins of the ubiquitin–proteasome system. Various ubiquitin C‐terminal hydrolases, E3 ubiquitin–protein ligases, subunits of the proteasome and proteasome interacting proteins as well as ubiquitin receptor and proteins involved in ubiquitin‐mediated proteolysis have been identified. Rpn13, a ubiquitin receptor that interacts with the dcTPR protein SGTA to facilitate degradation of cytosol mislocalized secretory and membrane proteins 9, is the only reported example of an interaction between dcTPR proteins and members of the ubiquitin–proteasome system via the dicarboxylate clamp mechanism.

However, the anomalously high hit rate in our search indicates that regulation of protein metabolism via molecular chaperone‐mediated quality control as well as via the ubiquitin–proteasome system could be the general function of the dcTPR protein interaction network. The remaining proteins that possess the Hsp70/Hsp90‐like motif at their C termini could not be assigned to any functional subgroups. Among them, only Tom20 has been shown to interact with dcTPR proteins, Tom70 6 and AIP 7, via the dicarboxylate clamp mechanism. Some others, such as NADSYN1, phosducin‐like protein and DNAAF2, were identified from the molecular chaperone interactome 5 (Fig. 2); however, the mechanism of their interaction with molecular chaperones was not investigated. Another dcTPR protein, DYX1C1, also known as dynein axonemal assembly factor 4, was found associated with various cytoskeletal proteins including ones identified in our search, vimentin 21 and DNAAF2 22. Again, molecular mechanisms of the DYX1C1–vimentin and DYX1C1–DNAAF2 interactions were not investigated in detail. Many of the proteins identified in our global proteome search possess C termini highly similar to Hsp70 and Hsp90 making them good candidates for interaction with dcTPR proteins.

### *In vitro* and *in situ* interactions of Usp19, DNAAF2, NADSYN1, p23/PTGES3, Hsp105 and Bcl‐G with dcTPR proteins

**Figure 4:**
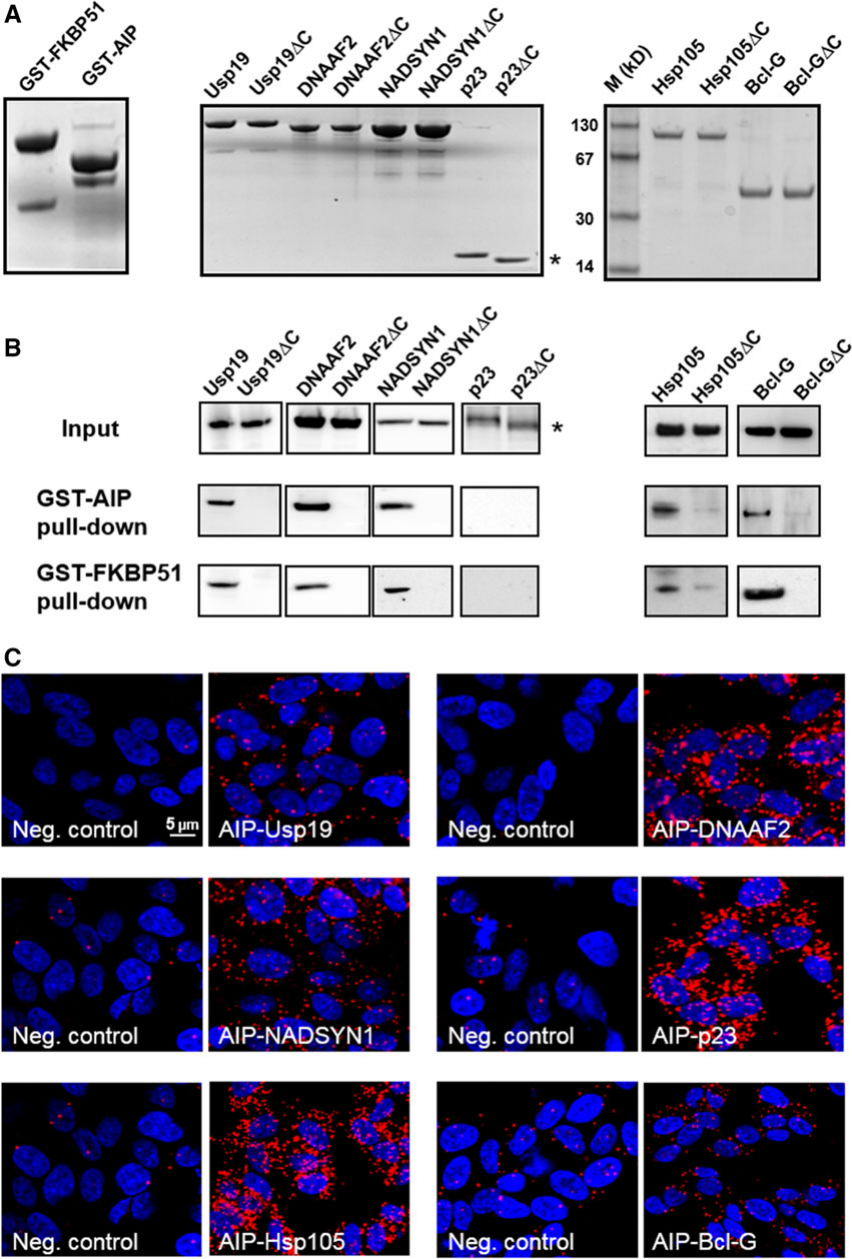
*In vitro* and *in situ* interactions of Usp19, DNAAF2, NADSYN1, p23/PTGES3, Hsp105 and Bcl‐G with dcTPR proteins. (A) Coomassie blue R staining of overexpressed and purified proteins used in GST pull‐down assay. (B) Western blot analysis of GST–AIP and GST–FKBP51 pull‐down of Usp19, DNAAF2, NADSYN1, p23/PTGES3, Hsp105, Bcl‐G and their truncated versions lacking five utmost C‐terminal residues. (C) PLA of Usp19, DNAAF2, NADSYN1, p23/PTGES3, Hsp105 and Bcl‐G interacting with AIP in HEK293 cells. The asterisk indicates position of truncated p23 on the gel.

To confirm dicarboxylate clamp interaction mechanism for several potential protein pairs that have been previously identified 5, we probed interactions of Usp19, DNAAF2, NADSYN1, p23, Hsp105 and Bcl‐G with the dcTPR proteins AIP and FKBP51. First, we overexpressed and purified GST, GST–AIP and GST–FKBP51 as well as Usp19, DNAAF2, NADSYN1, p23, Hsp105 and Bcl‐G and their truncated versions lacking the five utmost C‐terminal residues (Fig. 4A). GST pull‐down assay using GST–AIP and GST–FKBP51 as bait and full‐length and truncated Usp19, DNAAF2, NADSYN1, p23, Hsp105, and Bcl‐G as prey was performed and bound proteins were probed with their respective antibodies (Fig. 4B). While none of the assayed proteins interacted with GST alone (not shown), the pull‐down assay revealed that Usp19, DNAAF2, NADSYN1, Hsp105, and Bcl‐G interact with both AIP and FKBP51 and these interactions were dependent on the presence of an intact C terminus (Fig. 4B), confirming the importance of their C termini for interaction with dcTPR proteins. We could not, however, recover p23 in our pull‐down assay. Similar findings were reported previously 23 suggesting that p23 does not interact directly with dcTPR proteins.

To confirm respective protein–protein interactions *in situ*, we performed a PLA in HEK293 cells using specific antibodies (Fig. 4C). PLA is a technique used to detect protein–protein associations with very high specificity and sensitivity *in situ* 24. Because PLA detects protein–protein associations *in situ*, it avoids the risk of artifacts caused by overexpression of fluorescently tagged proteins used, for instance, in Förster resonance energy transfer experiments. PLA also allows quantification of signals upon various conditions and treatments. AIP interactions with Usp19, DNAAF2, NADSYN1, p23, Hsp105, and BclG are shown in Fig. 4C and FKBP51 interactions with these proteins were found to be similar to those of AIP (not shown). We obtained moderate to strong labeling for Usp19, DNAAF2, and NADSYN1 interactions with AIP and surprisingly strong labeling of AIP–p23 interactions. Similar results were obtained for FKBP51–p23 interactions (not shown). p23 is a Hsp90 co‐chaperone binding to the N‐terminal domain of Hsp90 independently of dcTPR co‐chaperones 25 as a part of a Hsp90–p23–dcTPR supercomplex, providing an explanation for previously observed interactions between p23 and various dcTPR proteins 6 and our PLA results. To test this, we probed with PLA the interactions of p23, NADSYN1, Hsp105, and Bcl‐G with Hsp90. Only the p23–Hsp90 antibody pair produced strong labeling supporting the Hsp90–p23–dcTPR supercomplex model (data not shown).

### Interaction of dcTPR proteins with 10‐amino‐acid peptides corresponding to C termini of dcTPR interaction partners

**Figure 5:**
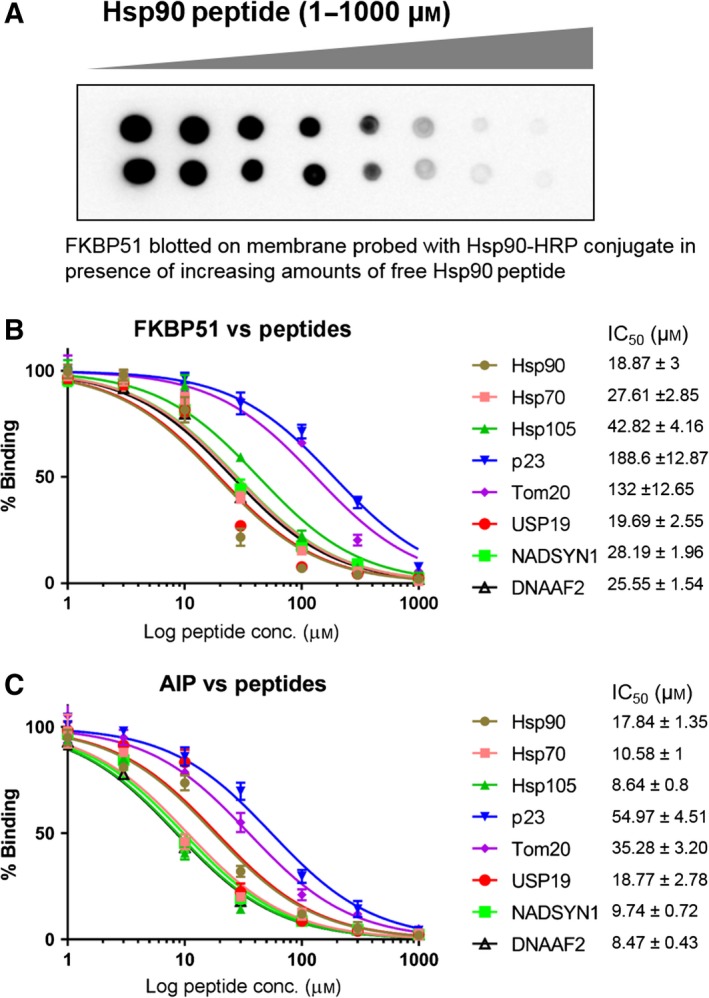
Analysis of interaction of FKBP51 and AIP with 10‐amino‐acid peptides corresponding to C termini of Hsp90, Hsp70, Hsp105, Tom20, and p23. (A) Dot‐blot competition assay of interactions between FKBP51 and Hsp90 peptide conjugated to HRP incubated with increasing amounts of unconjugated Hsp90 peptide. (B) Inhibition curves of FKBP51 interactions with HRP‐conjugated 10‐amino‐acid peptides corresponding to C termini of Hsp90, Hsp70, Hsp105, Tom20, and p23 probed with increasing concentrations of label‐free peptides. (C) As in (B) but AIP was used instead of FKBP51. *n* = 3. Graphs were created and SD values calculated using prism, version 7.02.

We analyzed interactions of the dcTPR proteins FKBP51 and AIP with several decapeptides corresponding to C termini of dcTPR partners, namely Hsp90β, Hsp70.1, Hsp105, p23 and Tom20 as well as poly‐D negative control peptide. Each peptide was separately coupled to HRP. FKBP51 was blotted onto nitrocellulose membrane and probed with HRP‐conjugated peptides (Fig. 5A). Addition of increasing amounts of free unconjugated peptides competing for the same peptide‐binding site resulted in a decrease of the luminescence signal (Fig. 5A). Inhibition curves for peptide interaction with FKBP51 and AIP are presented in Fig. 5B and C, respectively. While poly‐D HRP‐conjugated control peptide did not produce any signal in a dot‐blot binding assay other than for peptide–HRP conjugates that bind to immobilized dcTPR proteins with various affinities. For immobilized FKBP51, the estimated IC_50_ for Hsp90β‐derived peptide was 16 μm; for Usp19 peptide, 19 μm; for DNAAF2 peptide, 25 μm; for Hsp70.1 peptide, 26 μm; for NADSYN1 peptide, 28 μm; for Hsp105 peptide, 40 μm; for Tom20 peptide, 183 μm; and for p23 peptide, 291 μm. For nitrocellulose‐immobilized AIP, the estimated IC_50_ for Hsp90β peptide was 18 μm; for Usp19 peptide, 9 μm; for DNAAF2 peptide, 8 μm; for Hsp70.1 peptide, 7 μm; for NADSYN1 peptide, 18 μm; for Hsp105 peptide, 6 μm; for Tom20 peptide, 62 μm; and for p23 peptide, 47 μm (Fig. 5B,C). These results indicate more than 10‐fold differences in binding strength between peptides derived from Tom20 or p23 and peptides derived from other dcTPR proteins. One plausible explanation of variations in the binding strength between these peptides would be the presence of C‐terminal glutamates in Tom20 and p23 peptides that form sterically unfavorable interactions with the dcTPR residues.

### Selective disruption of dcTPR protein interactions with their partners containing Hsp70/Hsp90‐like C termini using cell‐permeant peptides containing acidic C‐terminal sequences

**Figure 6:**
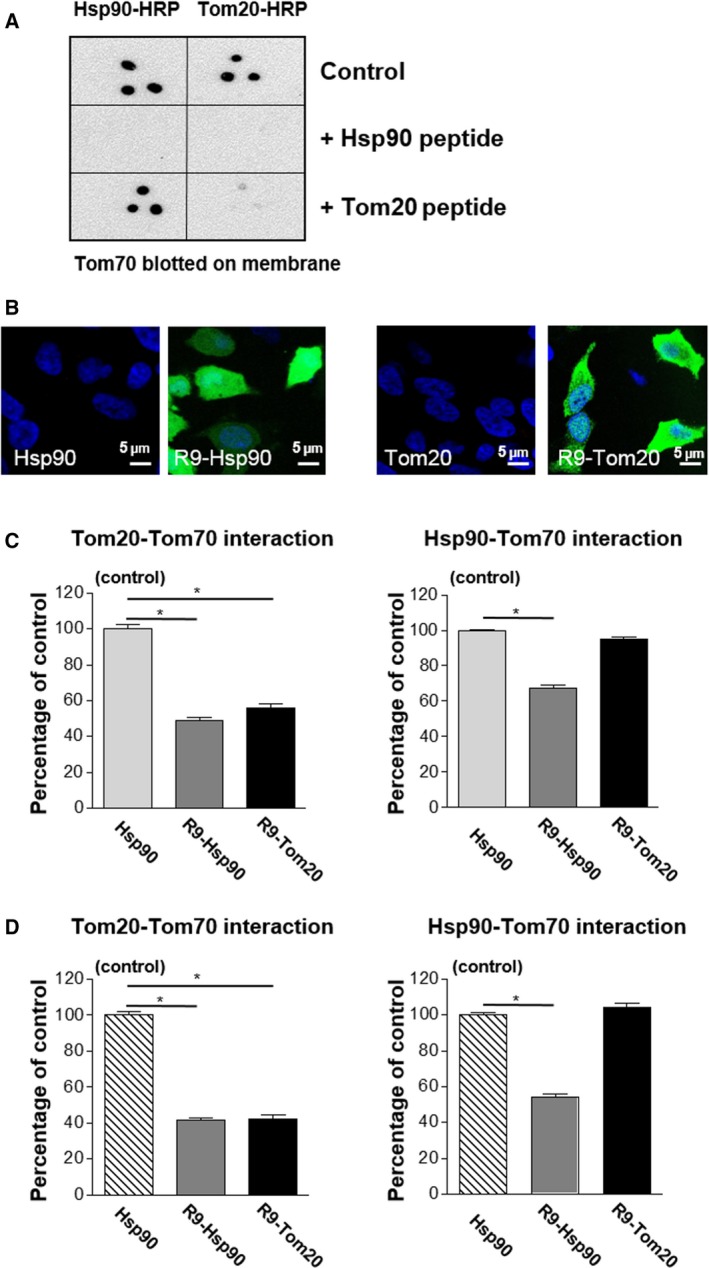
*In vitro* and *in situ* analysis of interactions of 10‐amino‐acid peptides corresponding to C termini of Hsp90 and Tom20 with Tom70. (A) Dot‐blot assay of interactions between Tom70 and HRP‐conjugated 10‐amino‐acid peptides corresponding to C termini of Hsp90 and Tom20 in the presence of 20 μm of unconjugated peptides. (B) Fluorescence imaging of HEK293 cells in the presence of 20 μm of fluorescein‐labeled Hsp90, R9–Hsp90, Tom20 and R9–Tom20 peptides. (C) Inhibition of Tom20–Tom70 and Hsp90–Tom70 interactions with R9–Hsp90 and R9–Tom20 in HEK293 cells measured by PLA. *n* = 3. Graphs were created and SD values were calculated using prism, version 7.02. *P‐value < 0.05 compared to control; comparisons are based on one‐way ANOVA. (D) Inhibition of Tom20–Tom70 and Hsp90–Tom70 interactions with R9–Hsp90 and R9–Tom20 in HEK293 cells measured by mammalian two‐hybrid assay. *n* = 3. Graphs were created and SD values were calculated using prism, version 7.02. *P‐value < 0.05 compared to control; comparisons are based on one‐way ANOVA.

It has been previously shown that a 10‐amino‐acid peptide corresponding to C termini of Hsp90 has more than 10‐fold higher affinity for the dcTPR protein Tom70 than for 10‐mer Tom20 peptide 6. First, we tested the ability of unlabeled Hsp90 and Tom20 peptides to compete with their HRP‐labeled analogues for binding to Tom70, the known partner for both Hsp90 and Tom20 proteins. Dot‐blot experiments revealed that while Hsp90 unlabeled peptide effectively competes for Tom70 binding with both HRP‐conjugated Hsp90 and Tom20 peptides, Tom20 label‐free peptide is unable to disrupt Tom70's interaction with HRP–Hsp90 (Fig. 6A). To evaluate efficiency and selectivity of peptides in disruption of protein interactions within the dcTPR network *in situ*, we treated HEK293 cells with 10‐amino‐acid peptides corresponding to the C termini of Hsp90 and Tom20 proteins as well as their cell‐permeant analogs containing a nine‐arginine N‐terminal tag (R9–Hsp90 and R9–Tom20, respectively). We labeled these peptides N‐terminally with fluorescein and observed that only cell‐permeant peptides, R9–Hsp90 and R9–Tom20, resulted in HEK293 cells staining upon addition of 20 μm peptides (Fig. 6B). At this peptide concentration, treated HEK293 cells did not show signs of cell death after 24 h of treatment (data not shown). Cytoplasmic fluorescein staining with occasional punctate cytoplasmic inclusions was observed. No fluorescein staining of peptides without the nine‐arginine tag was visible in treated cells. These results indicate that the R9 tag is efficient in peptide translocation into the cells. In subsequent experiments, non‐fluorescent peptides were used. We performed PLA for Tom20‐Tom70 and Hsp90‐Tom70 interactions using respective antibodies and quantified staining in absence or presence of cell‐permeable R9–Hsp90 and R9–Tom20 peptides (Fig. 6C). Cell impermeable Hsp90 peptide served as a treatment control. The results indicated that while R9–Hsp90 peptide addition decreased Tom20–Tom70 and Hsp90–Tom70 interaction signals by approximately 50%, addition of R9–Tom20 peptide failed to inhibit Hsp90–Tom70 interaction (Fig. 6C). The results suggested a hierarchal mode of interaction within the dicarboxylate clamp network – peptides with low affinity for the dicarboxylate clamp cannot disrupt high‐affinity interactions. Further, to confirm our initial findings, we performed a mammalian two‐hybrid system assay and studied the effect of peptides addition. Tom20 and Hsp90 were fused to a GAL4 DNA binding domain, and Tom70 was fused to a V16 activation domain, and luciferase expression was monitored via luminescence counting. After plasmid transfection, cells were treated with non‐permeant Hsp90 peptide that served as control and cell‐permeant R9–Hsp90 and R9–Tom20 peptides. The results of the mammalian two‐hybrid assay showed that the Tom20–Tom70 interaction was sensitive to addition of both R9–Hsp90 and R9–Tom20 peptides, whereas the Hsp90–Tom70 interaction was inhibited only by addition of R9–Hsp90 peptide (Fig. 6D). These results suggest that dcTPR protein interactions can be modulated *in situ* by addition of cell‐permeant Hsp70/Hsp90‐like peptides. Importantly, addition of peptides with low affinity towards the dcTPR domain preserves high‐affinity protein–protein interactions within the dcTPR network.

## Discussion

In the present article, we have for the first time described a comprehensive protein–protein interaction network comprising dcTPR proteins and proteins with Hsp70/Hsp90‐like acidic C termini. We identified 112 potential partners for dcTPR proteins in the human protein database. Functional analysis of the dcTPR proteins’ partners reveals clear over‐representation of proteins involved in a few distinct intracellular processes such as protein folding via the molecular chaperone system and protein degradation via the ubiquitin–proteasome system. We, in this article, and others confirmed interaction of particular members of the molecular chaperone family, proteins of the ubiquitin–proteasome system, with dcTPR proteins via the dicarboxylate clamp mechanism 5, 6, 7, 8.

The aim of this work was not to study the functional aspects of the interactions between the identified proteins, but to explore whether proteins containing a similar C terminus to that of Hsp90/Hsp70 can interact with the TPR‐containing proteins. We strongly believe that interactions between particular dcTPR proteins and proteins with Hsp70/Hsp90‐like acidic C termini listed in the Table S1 must be individually verified and assessed in a biological context, which can be considered as a limitation of this work. It has to be stressed that the list of proteins interacting via the dicarboxylate clamp mechanism is likely not complete and may, on the other hand, include proteins that do not interact with dcTPR proteins. Our search criteria could also miss a subset of dcTPR interaction partners. For example, it has been reported that patches of acidic residues resembling a Hsp70/Hsp90 C‐terminal motif buried within the protein primary sequence of Mcl‐1 can mediate interactions with dcTPR‐domain‐containing protein Tom70 via the dicarboxylate clamp mechanism making such motifs almost impossible to recognize by primary sequence search 26. Nevertheless, our results provide a global view of the dcTPR protein interaction landscape with important functional implications. Particular enrichment of proteins responsible for protein folding and degradation suggests functional specialization of proteins possessing Hsp70/Hsp90‐like C termini. Regarding the molecular chaperones, we have identified not only well‐known members of the Hsp70 and Hsp90 family, but also members of the Hsp105 family. Besides a single confirmatory report on interactions of Hsp104 with dcTPR proteins in yeast 8, no such data exist for the mammalian system. Based on our database search results as well as on peptide–protein interaction data (Fig. 4B,C), we believe that human proteins of the Hsp105 molecular chaperone family interact with dcTPR proteins by the dicarboxylate clamp mechanism. Proteins of ubiquitin proteasomal system regulate protein homeostasis in cytosol by degradation of proteins that are damaged or unable to fold correctly, and are also involved in cell development, cell signaling and stress response 27, 28. They co‐operate with molecular chaperones to control protein degradation in the cytosol.

We confirmed interaction of ubiquitin carboxypeptidase Usp19 with two dcTPR proteins, AIP and FKBP51, via the dicarboxylate clamp mechanism. Interestingly, Usp19 possess CS (CHORD and SGT1)/p23 domains in the N terminus that mediate its interaction with Hsp90 29. This suggests that the Hsp90–Usp19 complex can simultaneously mobilize two dcTPR proteins via their MEEVD C termini. Whether such a complex exists in the human cells and its composition and function remain to be determined. p23 also contains a CS (CHORD and SGT1) domain that mediates its interaction with Hsp90 30. p23 has been reported to interact with several dcTPR proteins 5; however, in our study we could not detect direct interactions of p23 with AIP or FKBP51 (Fig. 3B). Peptide competition experiments revealed that the p23 C‐terminal peptide has low affinity towards AIP and FKBP51 (Fig. 4B,C). Hypothetically, a Hsp90–p23 complex can provide two binding sites for dcTPR co‐chaperones, the high‐affinity site of the MEEVD C terminus of Hsp90 and the low‐affinity site of the p23 C terminus. The existence of two dcTPR protein binding sites in the Hsp90–p23–dcTPR super‐complex can provide the molecular basis for dcTPR co‐chaperone exchange upon hormone treatment 31. We found that peptides derived from Hsp70 and Hsp90 C termini have higher affinity for dcTPR proteins than peptides derived from C termini of Tom20 and p23. This provides an opportunity for selective disruption of low‐affinity interactions within the dcTPR protein interaction network as demonstrated in Fig. 5. Since some of the low‐affinity interactions can be of biological importance, cell‐permeant peptides or peptidomimetics structurally resembling C termini of low‐affinity partners for dcTPR proteins can be used for functional assessment of low‐affinity interactions as well as potential drug candidates. Analysis of available literature data supports the importance of an acidic C‐terminal Hsp70/Hsp90‐like motif for the protein's function. For example, two isoforms of Bcl‐2 protein family member Bcl‐G exist in humans 32. The short isoform, Bcl‐G_S_, is C‐terminally truncated in comparison to the longer Bcl‐G_L_ isoform. Bcl‐G short and long isoforms differ in their subcellular localization and in their ability to promote apoptosis 32. Another example of the functional importance of the Hsp70/Hsp90‐like motif at the C terminus is RNF‐14 (ARA54), which is a ubiquitin E3‐ligase that has been implicated in androgen receptor‐mediated signaling 33, 34. Miyamoto *et al*. 35 have identified the dominant negative mutant of ARA54 inhibiting androgen receptor‐mediated prostate cancer cell growth. The only difference from the non‐mutant ARA54 sequence was a point mutation within the Hsp70/Hsp90‐like motif at the C terminus changing a glutamate residue to lysine 35. These data warrant further investigations into whether the function of Bcl‐G and ARA54 is regulated by their interactions with dcTPR proteins.

Finally, in the present article we have explored the comprehensive protein–protein interaction network consisting of dcTPR proteins and proteins containing Hsp70/Hsp90‐like C termini that interact via the dicarboxylate clamp mechanism. Due to the abundance of cytosolic molecular chaperones of the Hsp70 and Hsp90 families and their relatively high affinity for dcTPR proteins, we believe that these proteins are dominant partners for dcTPR proteins. However, many proteins identified in this work possess C‐terminal sequences identical or very similar to that of Hsp70 and Hsp90 and can form relatively high‐affinity complexes with dcTPR proteins. On the other hand, low‐affinity interactions can also exist due either to additional contacts outside of C‐terminal residues or by physical proximity as has been suggested for Tom20–Tom70 interactions 6.

## Author contributions

AB contributed to planning experiments, analyzing data and manuscript preparation, RK contributed to performing experiments and manuscript preparation, BW contributed to experimental planning and manuscript preparation, PP contributed to planning and performing experiments, analyzing data, and manuscript preparation.

## Conflict of interest

The authors declare no conflict of interest.

## Supporting information


**Table S1.** List of identified human proteins that possess C‐terminal sequences similar to that of Hsp70/Hsp90 molecular chaperones.

**Table S2.** Gene ontology biological process obtained from GOrilla server.
