# Supplementary material for: In silico identification and biochemical characterization of the human dicarboxylate clamp TPR protein interaction network
Source: FEBS Open Bio. 2018 Oct 9;8(11):1830–43. doi: 10.1002/2211-5463.12521 (PMC6212638; doi:10.1002/2211-5463.12521)
Supplement: Supplementary file 1 — Table S1. List of identified human proteins that possess C‐terminal sequences similar to that of Hsp70/Hsp90 molecular chaperones. [file FEB4-8-1830-s001.docx]

**Table 1. List of identified human proteins that possess C-terminal sequences similar to that of Hsp70/Hsp90 molecular chaperones.**

| **Protein name** | **Accession №** | **C-terminal 5 a. a.** | **Length (a. a)** | **Function** |
| --- | --- | --- | --- | --- |
|  |  |  |  | **Molecular chaperone/co-chaperone** |
| Hsp90 alpha  Hsp90 beta  Hsp70.1  Hsp70.2  Hsp70.6  Hsp70.8, Hsc71  Hsp105  Hsp70-4  Hsp70-4L  P23  DNAJC16 | [AAI21063.1](https://www.ncbi.nlm.nih.gov/nuccore/115527971)  NP_001258899  AAD21816.1  NP_068814.2  NP_002146.2  NP_006588.1  NP_006635  NP_002145.3  NP_055093.2  Q15185  NP_001274740 | -MEEVD  -MEEVD  -IEEVD  -IEEVD  -IEEVD  -IEEVD  -NMDLD  -EMDID  -EMEVD  -MPDLE  -WPELD | 732  724  641  639  643  646  858  840  839  160  470 | Molecular chaperone, Hsp90 family  Molecular chaperone, Hsp90 family  Molecular chaperone, Hsp70 family  Molecular chaperone, Hsp70 family  Molecular chaperone, Hsp70 family  Molecular chaperone, Hsp70 family  Molecular chaperone, Hsp105 family  Molecular chaperone, Hsp105 family  Molecular chaperone, Hsp105 family  Co-chaperone of Hsp90  Co-chaperone of Hsp70 |
|  |  |  |  | **Ubiquitin proteosomal system** |
| Usp19 isoform  USP22  RNF14, ARA54  NEDD4  HERC1, isoform X8  SHPRH  TRAF6, isoform X1  PSMB10  ECM29  GET4, TRC35  SH3D21, isoform X7  ZFAND1, AN1-type zinc finger protein 1  TRIM39  ADRM1, RPN13  CUL9 | NP_001186090  Q9UPT9  Q9UBS8  P46934  XP_016878195  Q149N8  XP_016873709  P40306  Q5VYK3  Q7L5D6  XP_011540455  Q8TCF1  Q9HCM9  Q16186  Q8IWT3 | -MEEVD  -FLEYE  -DEVED  -FEGVD  -VEAVD  -TEELE  -ETEID  -AMEVE  -LENLE  -PIELD  -EVQLE  -ESYLE  -PTDWE  -DMSLD  -DEAYD | 1384  525  474  1319  2792  1683  324  273  1845  327  712  268  518  407  2517 | Ubiquitin carboxy-terminal hydrolase  Ubiquitin carboxy-terminal hydrolase  E3 ubiquitin-protein ligase, androgen receptor activator  E3 ubiquitin-protein ligase  E3 ubiquitin-protein ligase  E3 ubiquitin-protein ligase  E3 ubiquitin-protein ligase, TNF receptor-associated factor  Proteosome subunit  Proteosome associated protein  Protein targeting and quality control  Contains ubiquitin interaction domain  Ubiquitin mediated proteolysis  E3 ubiquitin-protein ligase  Ubiquitin receptor, adhesion regulating molecule  Core component of a Cul9-RING ubiquitin-protein ligase complex |
| VIM, Vimentin  DNAAF2, Kintoun  WASF1  DRC3  CORO7  RSPH4A  KIZ  YPEL1  YPEL4  YPEL2  YPEL3  ZMYM3, ZNF261  BRD1  NSD3, isoform  PRDM6  SETD2, HIP1  KCTD1  KCTD15  FOXP2  ZMYND8  RAD26L, isoform  RAD9B, isoform X1  RDM1  PURG isoform a  DDX4  DDX60 isoform X4  DHX58  LARP7  APOBEC3F  HDGFRP3, isoform X1  TCF25  PKNOX2  MND1  SHPRH  DNA polymerase epsilon subunit 4  ABHD4  ABHD5  ABHD2  Lipin3, LPIN3  DES1  Perilipin 2, isoform X1  CCDC91, p56  BICD1, isoform X1  TXLNB, Taxilin beta  TXLNG, Taxilin gamma  SORT1, Sortilin 1  PHD, Phosducin  PHLP, phosducin-like  GPR65  Opsin 5, Neuropsin  GRP179  Tom20  MSTO1, Misato 1  BCLG, Bcl2-like 14  FKBP3, FKBP25  CYP2F1, isoform X3  ACVR1B  CDCA5, soronin  PGM5, isoform X2  IKKAP1, NEMO  NADSYN1  LXN, Latexin  Caskin1  Caskin2  TXNDC15  TXNDC6  TXL-2  SLC39A8  SLC2A13  CDKL3 isoform X5  PCDH1  Protocadherin β 15  RTL1, PEG11  OR11A1  AGTR1  Parvalbumin isoform CRA_b  U68  GMPS  NME2  P4HA2  NAA20, NAT5  RPP14  DCDC2C  BSDC1  TEX44 | NP_003371  Q9NVR5  Q92558  Q9H069  P57737  Q5TD94  Q2M2Z5  O60688  Q96NS1  Q96QA6  P61236  Q14202  CAG30294  NP_060248  Q9NQX0  Q9BYW2  Q719H9  BAG57750  O15409  Q9ULU4  EAW92637  XP_011536273  Q8NG50  NP_037489  Q9NQI0  XP_016863873  Q96C10  Q4G0J3  Q8IUX4  XP_006720617  Q9BQ70  Q96KN3  Q9BWT6  AAI17687  NP_063949  Q8TB40  Q8WTS1  P08910  Q9BQK8  O15121  XP_016869748  XP_016875059  XP_011519114  AAI15384  Q9NUQ3  Q99523  P20941  Q13371  Q8IYL9  Q6U736  Q6PRD1  Q15388  Q9BUK6  Q9BZR8  Q00688  XP_011524853  AAA60556  Q96FF9  XP_011517085  Q9Y6K9  Q6IA69  NP_064554  NP_065815  AAL49757  Q96J42  EAW79074  Q86XW9  Q9C0K1  Q96QE2  XP_016865019  Q08174  AAH38797  A6NKG5  NP_039225  AAH22447  EAW60119  AMD82198  P49915  P22392  O15460  P61599  O95059  A8MYV0  Q9NW68  NP_689827 | -HDDLE  -LYDLD  -VDWLD  -GDILD  -EDEWD  -EDDYD  -DDFYD  -DNGWE  -DNGWD  -DNGWD  -DNGWD  -EEDLD  -LSDID  -EETVD  -SIEVD  -DTELE  -QEPLD  -QEPLD  -SEDLE  -DTFWD  -QDVLE  -EFGVE  -LPELD  -QECLD  -DESWD  -TDHVD  -DLSLD  -FSEYD  -QEILE  -IIEID  -EGEWD  -SDSLE  -TDYID  -TEELE  -EGTLD  -CDSVD  -CDTVD  -EADLE  -LDTLD  -EMVLE  -GQDVE  -PVDIE  -DVQLD  -LEGVD  -IESVD  -EDLLE  -EEDVE  -DLEID  -LEVLE  -HEEWE  -PWDWE  -EDDVE  -DSLVD  -HEEVD  -LVDID  -GGEVD  -DQFVE  -DLLVE  -DSILD  -MECIE  -LDGVD  -EVQLE  -DAMLE  -DAMLD  -QEHVE  -ITDLD  -PEDVD  -EIELE  -ASDVE  -LPDVE  -TEVWE  -SEFLE  -DANLD  -TETLD  -CFEVE  -PADVD  -STELE  -TTEWE  -DVWYE  -STEVD  -PEDIE  -ELVLD  -FEAVE  -WEDWE  -DPNYD | 366  837  559  523  925  716  673  119  127  119  119  1370  1058  645  595  2564  257  287  715  1186  672  410  284  347  724  1015  678  583  373  212  676  472  205  1683  117  342  349  425  851  323  445  432  988  684  528  831  246  301  337  354  2367  145  570  327  224  498  487  252  351  419  706  222  1431  1202  360  240  330  460  648  617  1060  768  1358  315  359  107  114  693  152  535  178  124  355  430  395 | **Other functions**  Intermediate filament protein  Dynein assembly factor  Regulation of actin polymerization  Dynein-associated complex protein  F-actin regulator, Golgi to endosome transport  Signal transduction between central pair of microtubules and dynein  Centrosome associated protein  Centrosome associated protein  Centrosome associated protein  Centrosome associated protein  Centrosome associated protein  Part of histone deacetylase complex  Histone acetylation  Histone-lysine N-methyltransferase  Histone-lysine N-methyltransferase  Histone-lysine N-methyltransferase  Huntingtin binding  Transcription repressor  Transcription regulator  Transcription factor  Transcription regulator  DNA/RNA helicase  DNA binding  DNA binding (potential)  DNA binding  RNA helicase  RNA helicase  RNA helicase  Part of small nuclear ribonucleoprotein complex  DNA deaminase  Hepatoma-derived growth factor, potential chromatin binding  Transcription factor  Transcription factor, homeobox protein  Binds to DNA, stimulates recombination  Suppressor of genomic instability  Part of DNA polymerase 2  Phospholipid metabolism  Phospholipid metabolism  Acylglycerol lipase  Phospholipid synthesis  Sphinolipid biosynthesis  Lipid metabolism  Trans-Golgi network accessory protein  Golgi-to-ER retrograde transport  Involved in intracellular vesicle traffic  Involved in intracellular vesicle traffic Vesicular transport  G-protein binding, signal transduction  G-protein binding, signal transduction  G-protein coupled psychosine receptor  G-protein coupled receptor  G-protein coupled receptor, glutamate subfamily  Mitochondrial protein targeting  Mitochondrial morphology regulation  Apoptosis regulator  Immunophilin, FK506 binding protein  Cytochrome P450, xenobiotic metabolism  TGF-βGF-chrome P450, xenobiotic receptor  Cell cycle, separation of sister chromatids  Glucuronidation, xenobiotic metabolism  NF-kappaB regulator  NAD biosynthesis  Tissue carboxypeptidase inhibitor, tumor suppressor  Interacts with Ca^2+^-calmodulin –dependent protein kinase CASK  Interacts with Ca^2+^-calmodulin –dependent protein kinase CASK  Thioredoxin domain containing protein  Thioredoxin domain containing protein  Testis and lung specific thioderoxin  Zinc transporter  Proton myo-inositol cotransporter  Cell cycle, cyclin dependent kinase like  Protocadherin, cell-cell contacts  Cell-cell contacts  Retrotransposon-derived gene  Olfactory receptor  Angiotensin II receptor, type 1  Calcium binding, involved in muscle relaxation  Human herpes virus 6A  Glutamine amidotransferase  Nucleotide diphosphate kinase  Collagen synthesis and modification  Component of N-acetyltransefase B  Tumor supressor  Doublecortin domain  Unknown function, BSD domain  Testis expressed, unknown function |
