# Supplementary material for: In silico identification and biochemical characterization of the human dicarboxylate clamp TPR protein interaction network
Source: FEBS Open Bio. 2018 Oct 9;8(11):1830–43. doi: 10.1002/2211-5463.12521 (PMC6212638; doi:10.1002/2211-5463.12521)
Supplement: Supplementary file 2 — Table S2. Gene ontology biological process obtained from GOrilla server. [file FEB4-8-1830-s002.docx]

**Table 2 Gene Ontology Biological Process obtained from GOrilla server.**

| **GO term** | **Description** | **P-value^a^** | **FDR q-value^b^** | **Enrichment**  **(N, B, n, b)^c^** | [**Genes**](http://cbl-gorilla.cs.technion.ac.il/GOrilla/fhpoq1kz/GOResultsPROCESS.html#genes_info) |
| --- | --- | --- | --- | --- | --- |
| GO:0006986 | Response to unfolded protein | 4.37E-10 | 8.39E-7 | 14.29 (100,7,7,7) | HSPA4L - heat shock 70kda protein 4-like HSPA2 - heat shock 70kda protein 2 HSP90AB1 - heat shock protein 90kda alpha (cytosolic), class b member 1 HSPA4 - heat shock 70kda protein 4 HSPA6 - heat shock 70kda protein 6 (hsp70b') HSPH1 - heat shock 105kda/110kda protein 1 HSPA8 - heat shock 70kda protein 8 |
| GO:0035966 | Response to topologically incorrect protein | 4.37E-10 | 4.2E-7 | 14.29 (100,7,7,7) | HSPA4L - heat shock 70kda protein 4-like HSPA2 - heat shock 70kda protein 2 HSP90AB1 - heat shock protein 90kda alpha (cytosolic), class b member 1 HSPA4 - heat shock 70kda protein 4 HSPA6 - heat shock 70kda protein 6 (hsp70b') HSPH1 - heat shock 105kda/110kda protein 1 HSPA8 - heat shock 70kda protein 8 |
| GO:0006457 | Protein folding | 6.44E-9 | 4.12E-6 | 10.94 (100,8,8,7) | PTGES3 - prostaglandin e synthase 3 (cytosolic) HSPA4L - heat shock 70kda protein 4-like HSPA2 - heat shock 70kda protein 2 HSP90AB1 - heat shock protein 90kda alpha (cytosolic), class b member 1 HSPA6 - heat shock 70kda protein 6 (hsp70b') HSPH1 - heat shock 105kda/110kda protein 1 HSPA8 - heat shock 70kda protein 8 |
| GO:0006458 | '*De novo*' protein folding | 3.72E-6 | 1.78E-3 | 12.50 (100,5,8,5) | PTGES3 - prostaglandin e synthase 3 (cytosolic) HSPA2 - heat shock 70kda protein 2 HSPA6 - heat shock 70kda protein 6 (hsp70b') HSPH1 - heat shock 105kda/110kda protein 1 HSPA8 - heat shock 70kda protein 8 |
| GO:0061077 | Chaperone-mediated protein folding | 3.72E-6 | 1.43E-3 | 12.50 (100,5,8,5) | PTGES3 - prostaglandin e synthase 3 (cytosolic) HSPA2 - heat shock 70kda protein 2 HSPA6 - heat shock 70kda protein 6 (hsp70b') HSPH1 - heat shock 105kda/110kda protein 1 HSPA8 - heat shock 70kda protein 8 |
| GO:0051085 | Chaperone cofactor-dependent protein refolding | 3.72E-6 | 1.19E-3 | 12.50 (100,5,8,5) | PTGES3 - prostaglandin e synthase 3 (cytosolic) HSPA2 - heat shock 70kda protein 2 HSPA6 - heat shock 70kda protein 6 (hsp70b') HSPH1 - heat shock 105kda/110kda protein 1 HSPA8 - heat shock 70kda protein 8 |
| GO:0051084 | '*De novo*' posttranslational protein folding | 3.72E-6 | 1.02E-3 | 12.50 (100,5,8,5) | PTGES3 - prostaglandin e synthase 3 (cytosolic) HSPA2 - heat shock 70kda protein 2 HSPA6 - heat shock 70kda protein 6 (hsp70b') HSPH1 - heat shock 105kda/110kda protein 1 HSPA8 - heat shock 70kda protein 8 |
| GO:0032446 | Protein modification by small protein conjugation | 5.44E-6 | 1.31E-3 | 4.35 (100,8,23,8) | TRAF6 - tnf receptor-associated factor 6, e3 ubiquitin protein ligase NEDD4 - neural precursor cell expressed, developmentally down-regulated 4, e3 ubiquitin protein ligase HERC1 - hect and rld domain containing e3 ubiquitin protein ligase family member 1 USP22 - ubiquitin specific peptidase 22 RNF14 - ring finger protein 14 CUL9 - cullin 9 SHPRH - snf2 histone linker phd ring helicase, e3 ubiquitin protein ligase TRIM39 - tripartite motif containing 39 |
| GO:0016567 | Protein ubiquitination | 5.44E-6 | 1.16E-3 | 4.35 (100,8,23,8) | TRAF6 - tnf receptor-associated factor 6, e3 ubiquitin protein ligase NEDD4 - neural precursor cell expressed, developmentally down-regulated 4, e3 ubiquitin protein ligase USP22 - ubiquitin specific peptidase 22 HERC1 - hect and rld domain containing e3 ubiquitin protein ligase family member 1 RNF14 - ring finger protein 14 CUL9 - cullin 9 SHPRH - snf2 histone linker phd ring helicase, e3 ubiquitin protein ligase TRIM39 - tripartite motif containing 39 |
| GO:0010033 | Response to organic substance | 7.84E-6 | 1.5E-3 | 5.88 (100,17,7,7) | HSPA4L - heat shock 70kda protein 4-like HSPA2 - heat shock 70kda protein 2 HSP90AB1 - heat shock protein 90kda alpha (cytosolic), class b member 1 HSPA4 - heat shock 70kda protein 4 HSPA6 - heat shock 70kda protein 6 (hsp70b') HSPH1 - heat shock 105kda/110kda protein 1 HSPA8 - heat shock 70kda protein 8 |
| GO:0016192 | Vesicle-mediated transport | 1.25E-5 | 2.17E-3 | 10.00 (100,10,5,5) | HSP90AB1 - heat shock protein 90kda alpha (cytosolic), class b member 1 HSPA2 - heat shock 70kda protein 2 HSPA6 - heat shock 70kda protein 6 (hsp70b') HSPH1 - heat shock 105kda/110kda protein 1 HSPA8 - heat shock 70kda protein 8 |
| GO:0070647 | Protein modification by small protein conjugation or removal | 1.3E-5 | 2.08E-3 | 3.62 (100,12,23,10) | TRAF6 - tnf receptor-associated factor 6, e3 ubiquitin protein ligase ADRM1 - adhesion regulating molecule 1 NEDD4 - neural precursor cell expressed, developmentally down-regulated 4, e3 ubiquitin protein ligase USP22 - ubiquitin specific peptidase 22 HERC1 - hect and rld domain containing e3 ubiquitin protein ligase family member 1 RNF14 - ring finger protein 14 PSMB10 - proteasome (prosome, macropain) subunit, beta type, 10 CUL9 - cullin 9 SHPRH - snf2 histone linker phd ring helicase, e3 ubiquitin protein ligase TRIM39 - tripartite motif containing 39 |
| GO:0071310 | Cellular response to organic substance | 2.48E-5 | 3.67E-3 | 14.29 (100,7,4,4) | HSP90AB1 - heat shock protein 90kda alpha (cytosolic), class b member 1 HSPA2 - heat shock 70kda protein 2 HSPA6 - heat shock 70kda protein 6 (hsp70b') HSPA8 - heat shock 70kda protein 8 |
| GO:0042221 | Response to chemical | 3.27E-5 | 4.48E-3 | 5.00 (100,20,7,7) | HSPA4L - heat shock 70kda protein 4-like HSPA2 - heat shock 70kda protein 2 HSP90AB1 - heat shock protein 90kda alpha (cytosolic), class b member 1 HSPA4 - heat shock 70kda protein 4 HSPA6 - heat shock 70kda protein 6 (hsp70b') HSPH1 - heat shock 105kda/110kda protein 1 HSPA8 - heat shock 70kda protein 8 |
| GO:0006810 | Transport | 3.83E-5 | 4.9E-3 | 6.25 (100,16,6,6) | HSPA2 - heat shock 70kda protein 2 HSP90AB1 - heat shock protein 90kda alpha (cytosolic), class b member 1 HSPA4 - heat shock 70kda protein 4 HSPA6 - heat shock 70kda protein 6 (hsp70b') HSPH1 - heat shock 105kda/110kda protein 1 HSPA8 - heat shock 70kda protein 8 |
| GO:0009057 | Macromolecule catabolic process | 3.98E-5 | 4.77E-3 | 4.35 (100,7,23,7) | NEDD4 - neural precursor cell expressed, developmentally down-regulated 4, e3 ubiquitin protein ligase ADRM1 - adhesion regulating molecule 1 KIAA0368 - kiaa0368 USP22 - ubiquitin specific peptidase 22 PSMB10 - proteasome (prosome, macropain) subunit, beta type, 10 CUL9 - cullin 9 HSPA8 - heat shock 70kda protein 8 |
| GO:0010604 | Positive regulation of macromolecule metabolic process | 4.71E-5 | 5.32E-3 | 4.00 (100,15,15,9) | TRAF6 - tnf receptor-associated factor 6, e3 ubiquitin protein ligase NEDD4 - neural precursor cell expressed, developmentally down-regulated 4, e3 ubiquitin protein ligase USP22 - ubiquitin specific peptidase 22 PTGES3 - prostaglandin e synthase 3 (cytosolic) RNF14 - ring finger protein 14 HSPA2 - heat shock 70kda protein 2 HSP90AB1 - heat shock protein 90kda alpha (cytosolic), class b member 1 HSPH1 - heat shock 105kda/110kda protein 1 HSPA8 - heat shock 70kda protein 8 |
| GO:0051173 | Positive regulation of nitrogen compound metabolic process | 4.71E-5 | 5.02E-3 | 4.00 (100,15,15,9) | TRAF6 - tnf receptor-associated factor 6, e3 ubiquitin protein ligase NEDD4 - neural precursor cell expressed, developmentally down-regulated 4, e3 ubiquitin protein ligase USP22 - ubiquitin specific peptidase 22 RNF14 - ring finger protein 14 PTGES3 - prostaglandin e synthase 3 (cytosolic) HSPA2 - heat shock 70kda protein 2 HSP90AB1 - heat shock protein 90kda alpha (cytosolic), class b member 1 HSPH1 - heat shock 105kda/110kda protein 1 HSPA8 - heat shock 70kda protein 8 |
| GO:0070887 | Cellular response to chemical stimulus | 6.52E-5 | 6.59E-3 | 12.50 (100,8,4,4) | HSPA2 - heat shock 70kda protein 2 HSP90AB1 - heat shock protein 90kda alpha (cytosolic), class b member 1 HSPA6 - heat shock 70kda protein 6 (hsp70b') HSPA8 - heat shock 70kda protein 8 |
| GO:0006950 | Response to stress | 6.66E-5 | 6.39E-3 | 4.55 (100,22,7,7) | HSPA4L - heat shock 70kda protein 4-like HSPA2 - heat shock 70kda protein 2 HSP90AB1 - heat shock protein 90kda alpha (cytosolic), class b member 1 HSPA4 - heat shock 70kda protein 4 HSPA6 - heat shock 70kda protein 6 (hsp70b') HSPH1 - heat shock 105kda/110kda protein 1 HSPA8 - heat shock 70kda protein 8 |
| GO:1900034 | Regulation of cellular response to heat | 7.14E-5 | 6.53E-3 | 12.50 (100,4,8,4) | PTGES3 - prostaglandin e synthase 3 (cytosolic) HSP90AB1 - heat shock protein 90kda alpha (cytosolic), class b member 1 HSPH1 - heat shock 105kda/110kda protein 1 HSPA8 - heat shock 70kda protein 8 |
| GO:0034605 | Cellular response to heat | 7.42E-5 | 6.47E-3 | 25.00 (100,3,4,3) | HSPA2 - heat shock 70kda protein 2 HSPA6 - heat shock 70kda protein 6 (hsp70b') HSPA8 - heat shock 70kda protein 8 |
| GO:0034620 | Cellular response to unfolded protein | 7.42E-5 | 6.19E-3 | 25.00 (100,3,4,3) | HSPA2 - heat shock 70kda protein 2 HSPA6 - heat shock 70kda protein 6 (hsp70b') HSPA8 - heat shock 70kda protein 8 |
| GO:0042026 | Protein refolding | 7.42E-5 | 5.93E-3 | 25.00 (100,3,4,3) | HSPA2 - heat shock 70kda protein 2 HSPA6 - heat shock 70kda protein 6 (hsp70b') HSPA8 - heat shock 70kda protein 8 |
| GO:0009408 | Response to heat | 7.42E-5 | 5.7E-3 | 25.00 (100,3,4,3) | HSPA2 - heat shock 70kda protein 2 HSPA6 - heat shock 70kda protein 6 (hsp70b') HSPA8 - heat shock 70kda protein 8 |
| GO:0035967 | Cellular response to topologically incorrect protein | 7.42E-5 | 5.48E-3 | 25.00 (100,3,4,3) | HSPA2 - heat shock 70kda protein 2 HSPA6 - heat shock 70kda protein 6 (hsp70b') HSPA8 - heat shock 70kda protein 8 |
| GO:0009893 | Positive regulation of metabolic process | 1E-4 | 7.1E-3 | 3.75 (100,16,15,9) | TRAF6 - tnf receptor-associated factor 6, e3 ubiquitin protein ligase NEDD4 - neural precursor cell expressed, developmentally down-regulated 4, e3 ubiquitin protein ligase USP22 - ubiquitin specific peptidase 22 PTGES3 - prostaglandin e synthase 3 (cytosolic) RNF14 - ring finger protein 14 HSPA2 - heat shock 70kda protein 2 HSP90AB1 - heat shock protein 90kda alpha (cytosolic), class b member 1 HSPH1 - heat shock 105kda/110kda protein 1 HSPA8 - heat shock 70kda protein 8 |

**^a^P-value** is the enrichment p-value computed according to the mHG or HG model. This p-value is not corrected for multiple testing of 1919 GO terms.
**^b^FDR q-value** is the correction of the p-value for multiple testing using the Benjamini and Hochberg (1995) method. Namely, for the i^th^ term (ranked according to p-value) the FDR q-value is (p-value * number of GO terms) / i.
**^c^Enrichment (N, B, n, b)** is defined as follows: N - is the total number of genes; B - is the total number of genes associated with a specific GO term; n - is the number of genes in the top of the user's input list or in the target set when appropriate; b - is the number of genes in the intersection; Enrichment = (b/n) / (B/N).
